# Research hotspots and trends in immunotherapy for cholangiocarcinoma: a bibliometric analysis (2014-2023)

**DOI:** 10.3389/fimmu.2024.1436315

**Published:** 2024-11-26

**Authors:** Xilin Qu, Qian Wang, Fengfeng Zhu, Hao Liang, Zhangtao Long, Yachen Wu, Mengliang Jiang, Zhaohai Liu, Xiaoming Dai, Zhu Zhu

**Affiliations:** ^1^ The First Affiliated Hospital, Department of Hepatobiliary Surgery, Hengyang Medical School, University of South China, Hengyang, Hunan, China; ^2^ The First Affiliated Hospital, Gynecology & Obstetrics and Reproductive Medical Center, Hengyang Medical School, University of South China, Hengyang, Hunan, China

**Keywords:** cholangiocarcinoma, immunotherapy, bibliometrics, hotspots, trend, knowledge map

## Abstract

**Background:**

Cholangiocarcinoma (CCA) is a malignant tumor of the gastrointestinal tract with a poor prognosis. Immunotherapy plays an important role in the treatment of CCA. This study aimed to investigate the research hotspots and trends in immunotherapy for CCA.

**Methods:**

The *Web of Science Core Collection* was searched for literature related to CCA immunotherapy research from January 1, 2014, to December 31, 2023, and features such as country, institution, authors, references, and keywords in the included literature were quantitatively and visually analyzed using the VOS viewer and CiteSpace software.

**Results:**

A total of 252 English publications published between 2014 and 2023 were included. The publications were mainly from China and the United States, with Fudan University being the institution that published the most papers. The highest number of publications came from Frontiers in Oncology. The most prolific authors were Jia Fan, Jian Zhou from China and Pa-Thai Yenchitsomanus from Thailand, while the Journal of Clinical Oncology ranked first in the number of citations among the co-cited journals. In recent years, the focus of research has shifted from “immune checkpoint” and “chemotherapy” to “immunotherapy combined therapy.” Currently, the research frontiers are “microenvironment,” “immune cells,” and “macrophages.”

**Conclusion:**

Our study analyzes the research hotspots and trends in CCA to provide a knowledge map of immunotherapy research, which will serve as a reference and direction for future research.

## 1 Introduction

Cholangiocarcinoma (CCA) is represented by a group of malignancies arising from the biliary epithelium that accounts for approximately 15% of all primary liver cancers and 3% of gastrointestinal malignancies (1). Anatomically, CCA can be categorized into intrahepatic cholangiocarcinoma, perihilar cholangiocarcinoma, and distal cholangiocarcinoma (2). During recent years, the global incidence and mortality of CCA have steadily increased, with a 22% rise in incidence and a 39% increase in mortality in the United States from 1979 to 2004 (3). In addition, the 5-year overall survival for CCA patients is only 10%, with a median survival of 24 months (4). Currently, surgical resection and liver transplantation are the only radical treatments available for early-stage patients (5). However, once diagnosed, patients with CCA are usually in the advanced stage, when effective radical surgery is no longer possible (6). Therefore, more effective strategies are needed to treat CCA.

Cancer immunotherapy, which is a treatment that utilizes the human immune system to kill cancer cells (7), has attracted much attention in refractory cancers due to its potent and long-lasting antitumor activity and low cytotoxicity (8–10). Various immunotherapy approaches are currently in use, including cancer vaccines, adoptive cell therapy (ACT), and immune checkpoint inhibitors (ICIs) (11). However, immunotherapy for CCA is still in its infancy (12). Cancer vaccines and ACT, typical representatives of personalized treatment, have not been well studied in clinical trials for CCA. Due to the lack of suitable antigen targets, their efficacy remains uncertain (13). Nowadays, immunotherapy research on CCA focuses primarily on ICIs (14). Several clinical trials have explored the efficacy of ICI monotherapy in patients with advanced CCA (15). Additionally, ICI-based combination therapies with chemotherapy or targeted therapies have gained increasing attention to improve response rates and outcomes (16). Nevertheless, more than half of CCA patients have immunologically cold tumors with low response rates to immunotherapy (17). Furthermore, it is noteworthy that ICIs are ineffective and are more likely to encounter resistance in unscreened patients (18). Recently, some reviews have summarized the latest immunotherapeutic approaches for treating CCA (12, 19). However, due to differences in reporting time and topic focus, understanding of the global research and research hotspots related to CCA immunotherapy have not been systematically and comprehensively reviewed. Bibliometrics is a research method that characterizes the quality and quantity of publications for researchers, evaluates the current status, and provides a reference for clinical medical research through the analysis of measurement indicators. However, to date, there has been no bibliometric analysis of CCA immunotherapy research.

Therefore, by performing a quantitative analysis of the development of CCA immunotherapy research, this study aimed to explore the current status and research hotspots in this field globally and to provide clinicians and researchers with a comprehensive understanding of this topic.

## 2 Materials and methods

### 2.1 Data retrieval

The *Web of Science Core Collection* (WoSCC) was systematically searched for publications related to immunotherapy for CCA from January 1, 2014, to December 31, 2023 on October 13, 2024. The search formula was as follows: (TI = (“cholangiocarcinoma” OR “bile duct cancer” OR “biliary tract cancer”) AND AB = (“cholangiocarcinoma” OR “bile duct cancer” OR “biliary tract cancer”)) AND (TI = (“immunotherapy” OR “immune checkpoint inhibitors” OR “PD-1 inhibitors” OR “CTLA-4 inhibitors” OR “T cell therapy” OR “cancer vaccines” OR “immune modulation” OR “durvalumab” OR “pembrolizumab” OR “nivolumab”) OR AB = (“immunotherapy” OR “immune checkpoint inhibitors” OR “PD-1 inhibitors” OR “CTLA-4 inhibitors” OR “T cell therapy” OR “cancer vaccines” OR “immune modulation” OR “durvalumab” OR “pembrolizumab” OR “nivolumab”)) NOT TI = (“case report”) AND LA = (English) AND DT = (Article OR Review) AND PY = (2014-2023). The titles, abstracts, and keywords of the selected articles were related to CCA immunotherapy.

### 2.2 Screening process

The inclusion criteria were as follows: (1) focused on immunotherapy for CCA; (2) written in English; (3) publication type limited to “article” or “review”; (4) derived from the WOS core collection; and (5) published between January 1, 2014, and December 31, 2023. The exclusion criteria were as follows: (1) studies not related to immunotherapy for CCA; (2) publication types that were not designated “article” or “review” (e.g., conference abstracts, case reports, etc.). The authors independently evaluated the full texts of all publications. If there were disagreements that could not be resolved, decisions were made under the guidance of the corresponding author (Z.Z). The data were exported in plain text format.

### 2.3 Variables and analysis

For the included publications, the following data were extracted: authors, countries, institutions, journals, and keywords. In this study, VOS Viewer (version 1.6.20) and CiteSpace (version 6.3.1) were used for data visualization to analyze the extracted data (20). Keywords with strong citation bursts were analyzed and visualized using CiteSpace to explore frontiers in CCA immunotherapy research (21). The 2023 edition of Journal Citation Reports (JCR) and Impact Factor (IF) were included in the analysis as key indicators of the scientific value of the study.

## 3 Results

### 3.1 Annual growth trend of publications on immunotherapy for CCA

**Figure 1:**
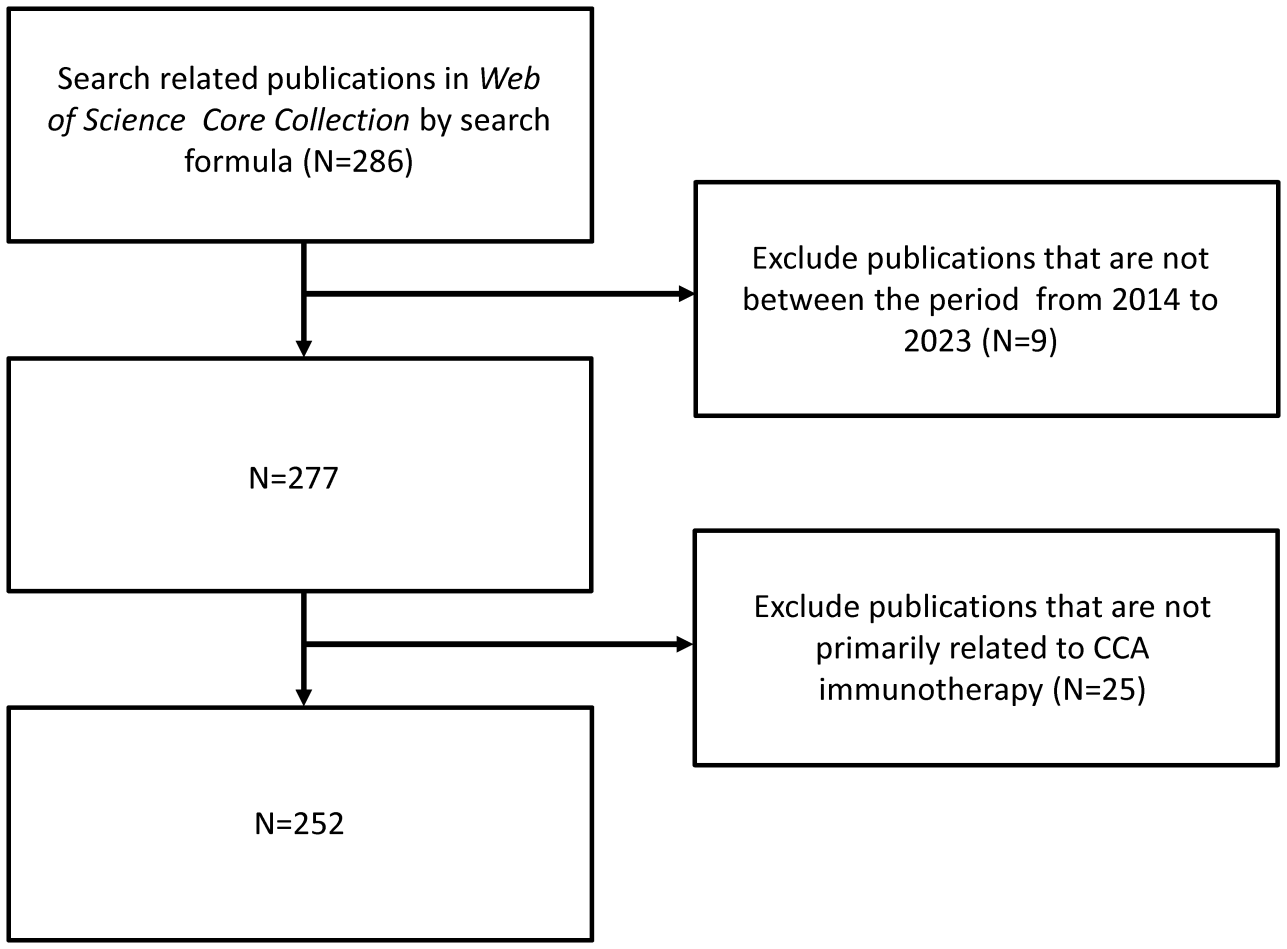
Flowchart of literature selection.

**Figure 2:**
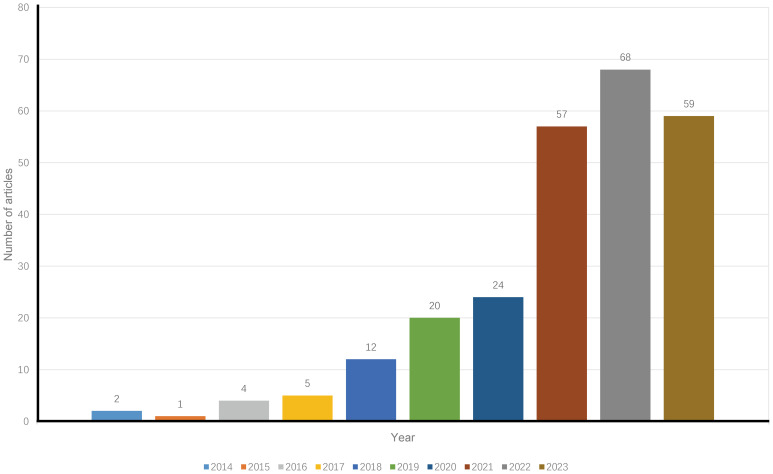
Annual trends of articles on immunotherapy for CCA published from 2014 to 2023.

A total of 252 publications related to immunotherapy for CCA were published from January 1, 2014, to December 31, 2023, including 199 articles (78.97%) and 53 reviews (21.03%). **Figure 1** shows a flow chart of the literature search. The research trend line was flat until 2020. After 2020, the number of annual publications increased rapidly, with 2022 representing the year with the highest number of publications (**Figure 2**).

### 3.2 Analysis of countries and institutions

**Figure 3:**
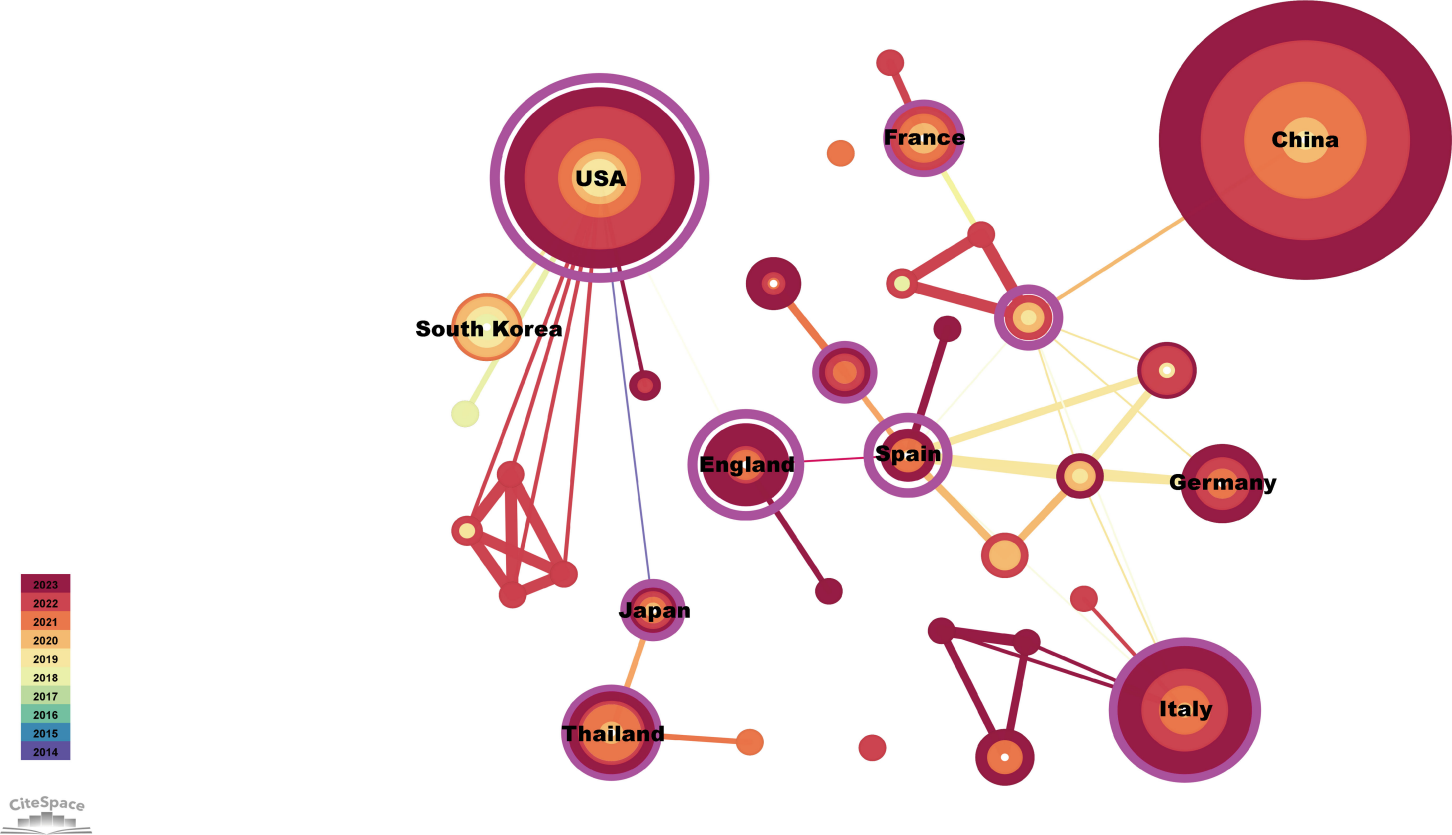
Visualization of countries publishing research related to immunotherapy for CCA. Cooperative network of publications between countries. Countries are represented by nodes. Partnerships are represented by lines. The node area increases with the number of publications. The colors represent different years.

The publications identified came from 34 countries (**Figure 3**). The countries with the most publications were China (n=123) and the United States (n=59), followed by Italy (n=27), Thailand (n=14), Germany (n=13), and England (n=11) (**Table 1**). Of the top 10 countries, seven have centrality greater than 0.1, with Spain and the United Kingdom being the highest at 1.04 and 0.85, respectively (**Table 1**). In addition, as the two countries with the most publications, China and the United States contributed to the use of immunotherapy for CCA from 2018 to 2023 (**Figure 3**).

**Table 1:** The top 10 most prolific countries.

Rank	Country	Centrality	Link strength	Count	Citations	Citation per articles
1	China	0.00	20	123	2189	17.80
2	USA	0.88	42	59	3293	55.81
3	Italy	0.40	27	27	782	28.96
4	Thailand	0.11	6	14	280	20.00
5	Germany	0.00	17	13	499	38.38
6	England	0.85	17	11	177	16.09
7	South Korea	0.00	1	9	364	40.44
8	France	0.11	4	8	426	53.25
9	Spain	1.04	17	6	351	58.50
10	Japan	0.21	4	6	95	15.83

**Figure 4:**
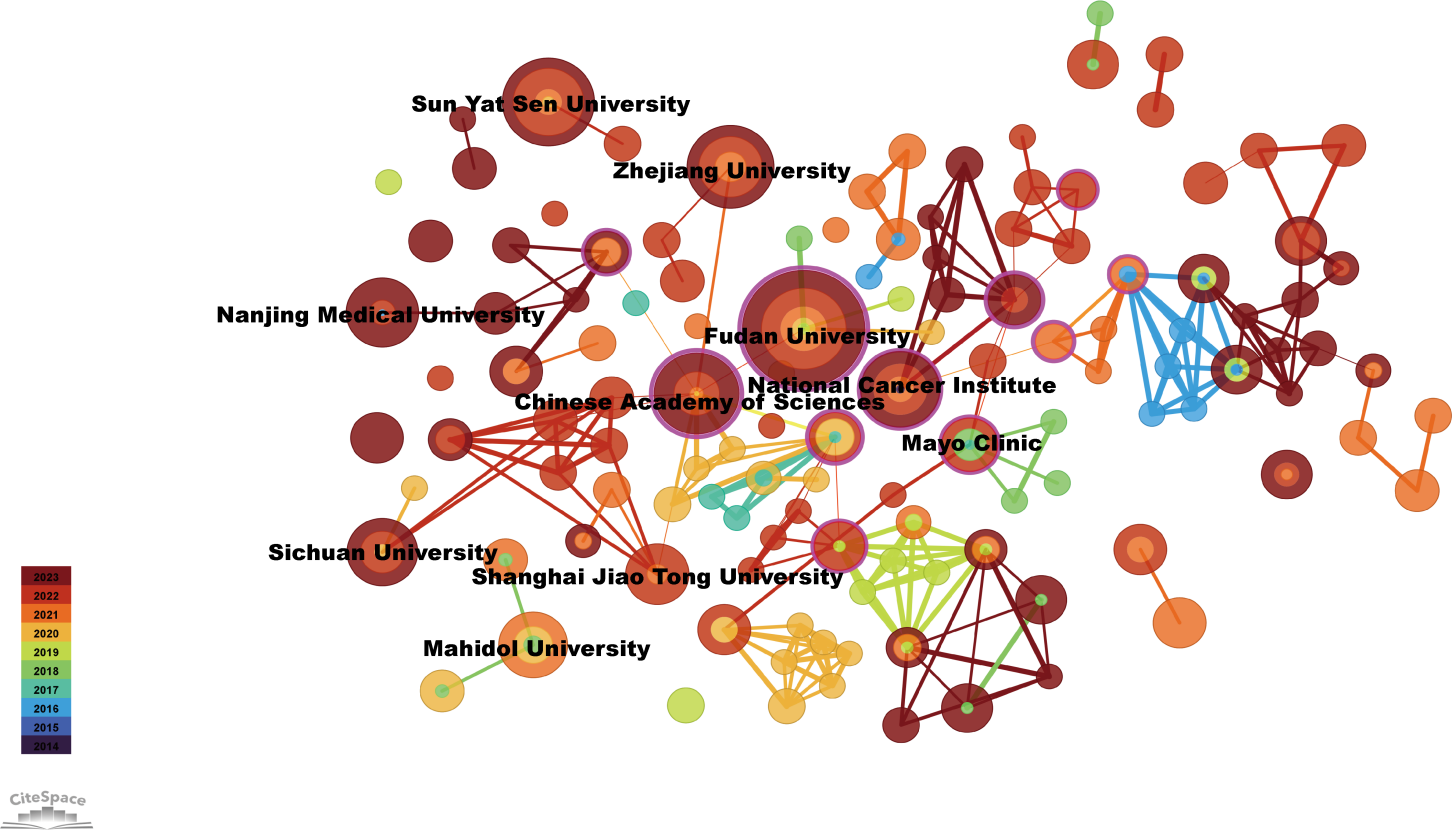
Visualization of institutions related to immunotherapy for CCA. Cooperative network of publications between institutions. Institutions are represented by nodes. Partnerships are represented by lines. The node area increases with the number of publications. The colors represent different years.

The top 10 institutions with the most publications were two from the United States, seven from China, and one from Thailand (**Figure 4**). Among them, Fudan University published 23 papers, followed by Sun Yat-Sen University with 11 (**Table 2**). The centrality of Mayo Clinic and National Cancer Institute from the United States, Fudan University and Shanghai Jiao Tong University from China were greater than 0.1, with values of 0.59, 0.11, 0.47 and 0.24, respectively (**Table 2**). In addition, among the top ten institutions, the National Cancer Institute had the most citations, with 1,714 (**Table 2**).

**Table 2:** The top 10 most prolific institutions.

Rank	Institution	Country	Centrality	Count	Citations	Citation per articles
1	Fudan University	China	0.47	23	825	35.87
2	Sun Yat Sen University	China	0.03	11	208	18.91
3	Zhejiang University	China	0.02	10	223	22.30
4	Mahidol University	Thailand	0.03	9	215	23.89
5	Mayo Clinic	USA	0.59	9	484	53.78
6	Nanjing Medical University	China	0.07	9	82	9.11
7	National Cancer Institute	USA	0.11	9	1714	190.44
8	Sichuan University	China	0.01	9	335	37.22
9	Chinese Academy of Sciences	China	0.00	8	291	36.38
10	Shanghai Jiao Tong University	China	0.24	7	365	52.14

### 3.3 Analysis of journals

**Figure 5:**
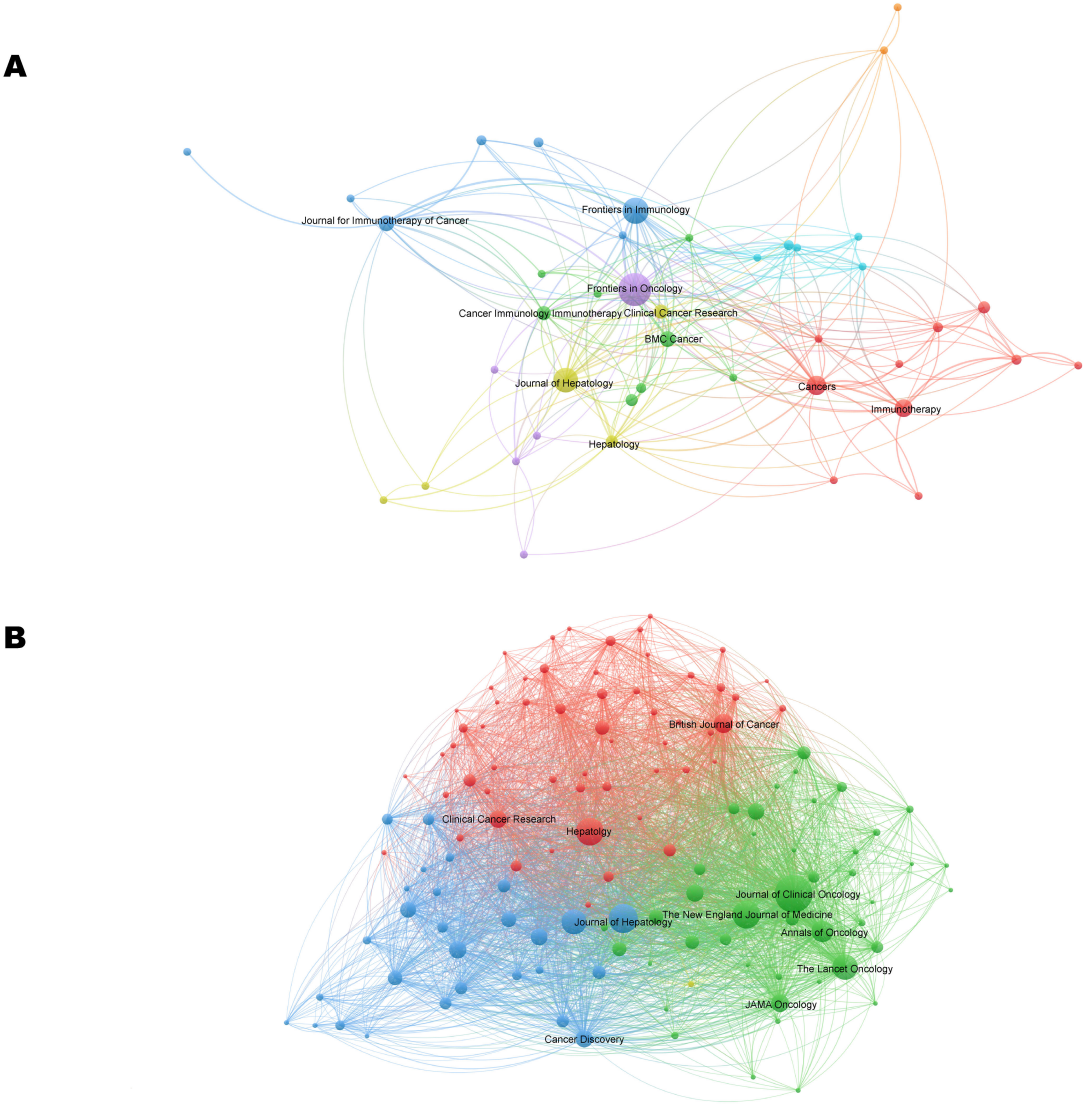
The visualization network of journals **(A)** and co-cited journals **(B)** related to CCA immunotherapy. The nodes with the same color represent the same cluster, implying a close partnership. The larger the node’s size or the width of the connecting line, the closer the relative degree of co-occurrence.

Publications in the field of immunotherapy for CCA were distributed across 133 journals. Of the top 10 most published journals, 70% were categorized as Q1, 20% were classified as Q2, and 10% were classified as Q3 (**Table 3**). The most published journals were Frontiers in Oncology (n=19), Frontiers in Immunology (n=13), and Journal of Hepatology (n=12) (**Figure 5A**; **Table 3**). Among the top 10 journals, the Journal of Hepatology (902 citations, IF=26.8) was also the journal with the highest number of citations and the highest impact factor (**Table 3**).

**Table 3:** Top 10 productive journals and co-cited journals in the field of CCA immunotherapy.

Rank	Journals	Count	Total citations	IF (2023)	JCR (2023)	Co-cited journals	Total citations	IF (2023)	JCR (2023)
1	Frontiers in Oncology	19	241	3.5	Q2	Journal of Clinical Oncology	650	42.1	Q1
2	Frontiers in Immunology	13	157	5.7	Q1	Journal of Hepatology	429	26.8	Q1
3	Journal of Hepatology	12	902	26.8	Q1	The New England Journal of Medicine	406	96.2	Q1
4	Cancers	8	251	4.5	Q1	Hepatolgy	397	12.9	Q1
5	Immunotherapy	7	30	2.7	Q3	The Lancet Oncology	352	41.6	Q1
6	BMC Cancer	6	86	3.4	Q2	Clinical Cancer Research	337	10	Q1
7	Journal for Immunotherapy of Cancer	6	322	10.3	Q1	Annals of Oncology	279	56.7	Q1
8	Cancer Immunology Immunotherapy	5	124	4.6	Q1	British Journal of Cancer	227	6.4	Q1
9	Clinical Cancer Research	5	229	10	Q1	JAMA Oncology	196	22.5	Q1
10	Hepatology	4	292	12.9	Q1	Cancer Discovery	195	29.7	Q1

**Figure 6:**
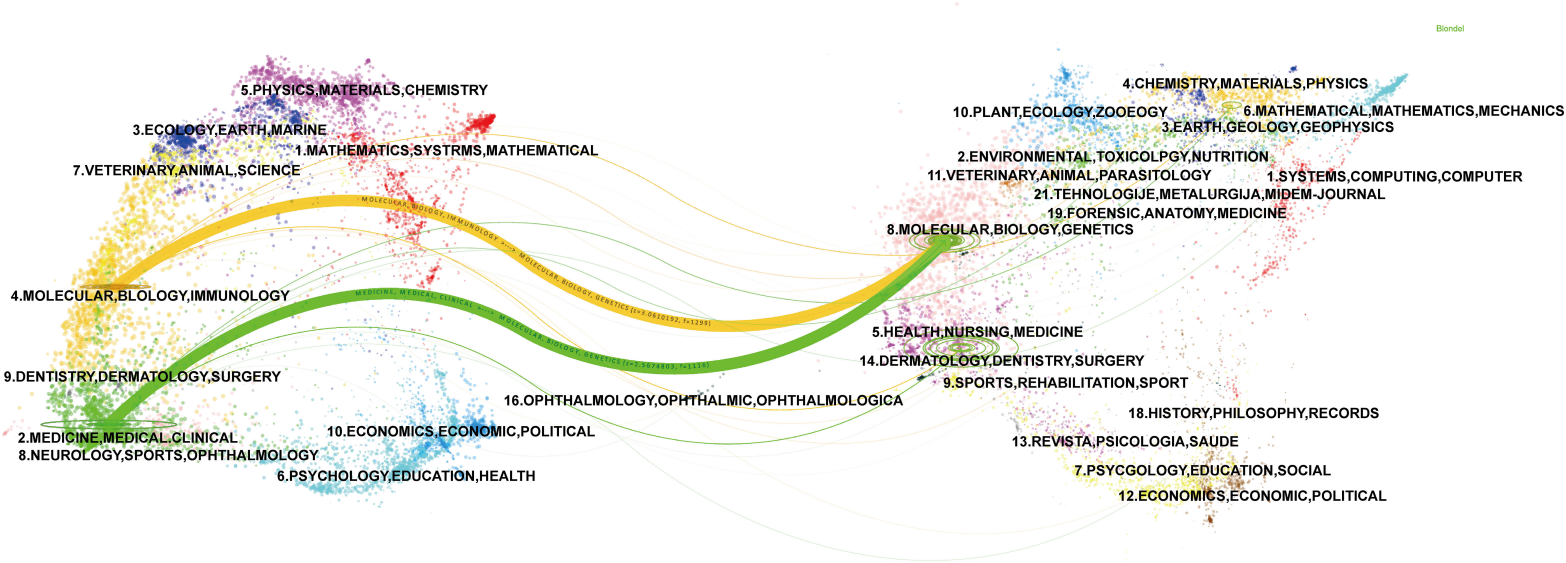
A dual-map overlay of journals related to immunotherapy for CCA. Citing journals are on the left, and the cited journals are on the right, with colored paths indicating citation relationships.

The most co-cited journal was the Journal of Clinical Oncology (650 citations), followed by Hepatology (429 citations) and the Journal of Hepatology (406 citations) (**Figure 5B**; **Table 3**). The top ten co-cited journals were all in Q1, with the New England Journal of Medicine having the highest IF (96.2) (**Table 3**). Dual-map overlay depicts the disciplinary distribution of academic journals, citation trajectories, and shifts in research centers (22). We found that research in molecular/biology/genetics was frequently cited by medical/clinical medicine journals in addition to being frequently cited by molecular/biology/immunology journals (**Figure 6**).

### 3.4 Analysis of authors

In the past decade, 1962 researchers have been involved in studies related to immunotherapy for CCA (**Supplementary Table S1**). The most prolific authors were Jia Fan and Jian Zhou from China and Pa-Thai Yenchitsomanus from Thailand with 9 articles, followed by Alessandro Rizzo (n=8), Giovanni Brandi (n=7) and Angela Dalia Ricci (n=7) from Italy, which showed quite similar citations. In addition, the most cited author was Alessandro Rizzo (330 citations). The most co-cited author was Juan Valle, who had 101 citations (**Supplementary Figure S1**). Jia Fan, Pa-Thai Yenchitsomanus, and Alessandro Rizzo were representative prolific authors from different countries, with the citation trajectories showing their key outputs in 2022, 2020, and 2021, respectively (**Supplementary Figure S2**). Based on the research theme, one cluster of authors mainly concentrated on studying the molecular mechanisms of immunotherapy for CCA, while the other predominantly centered on investigating therapeutic strategies through immunotherapy clinical trials.

### 3.5 Analysis of co-cited references and timeline

**Figure 7:**
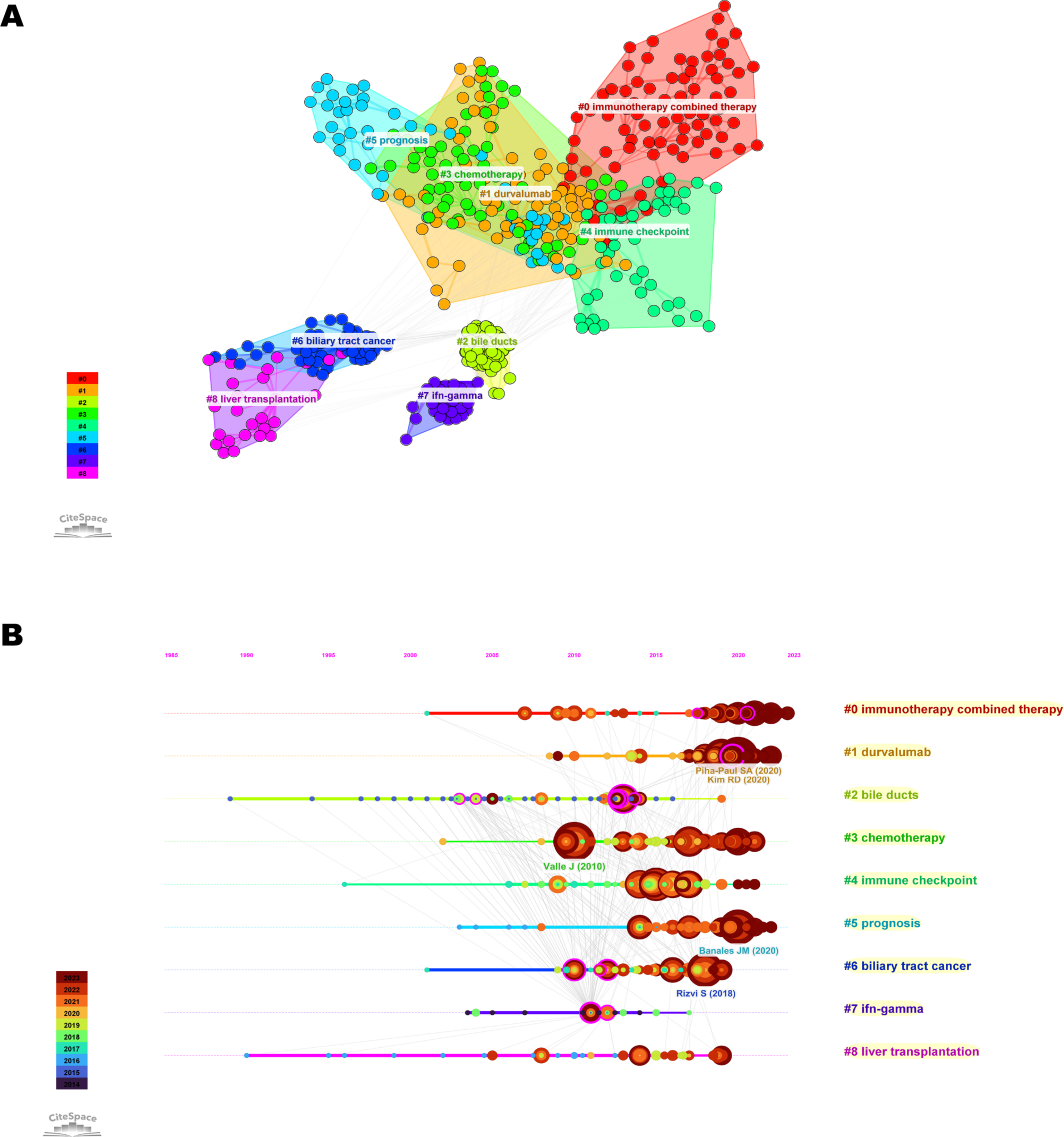
Visualization of co-cited references related to immunotherapy for CCA. **(A)** Knowledge map of co-cited references. Different colors represent different clusters. **(B)** Timeline view of co-cited references related to immunotherapy for CCA. The position of the node on the horizontal axis represents the time when the reference first appeared. The size of the node is related to the number of co-cited references. Lines between nodes represent co-cited relationships.

Co-cited literature is defined as publications cited by other authors and is considered to represent the knowledge base of a field (20). The co-cited literature was divided into 9 clusters (**Figure 7A**). Among them, five clusters, namely “immunotherapy combined therapy,” “durvalumab,” “chemotherapy,” “immune checkpoint,” and “prognosis” were closely related. The top co-cited reference was the article entitled “Cisplatin plus gemcitabine versus gemcitabine for biliary tract cancer” from the New England Journal of Medicine (IF=96.2), which were cited a total of 78 times (**Supplementary Table S2**).

Timeline view is a method of data visualization that combines clustering and time-slice techniques to show trends and interrelationships of research topics over time, in addition to illustrating the distribution of issues in the field (23). The most frequent words in each cluster are identified as cluster labels. Therefore, we plotted a timeline view of the co-cited literature (**Figure 7B**). We found that “chemotherapy” and “immune checkpoint” were the early research priority of immunotherapy for CCA, while “immunotherapy combined therapy” was the newer research priority that primarily developed from the former.

### 3.6 Analysis of keyword co-occurrence clustering and burst

**Figure 8:**
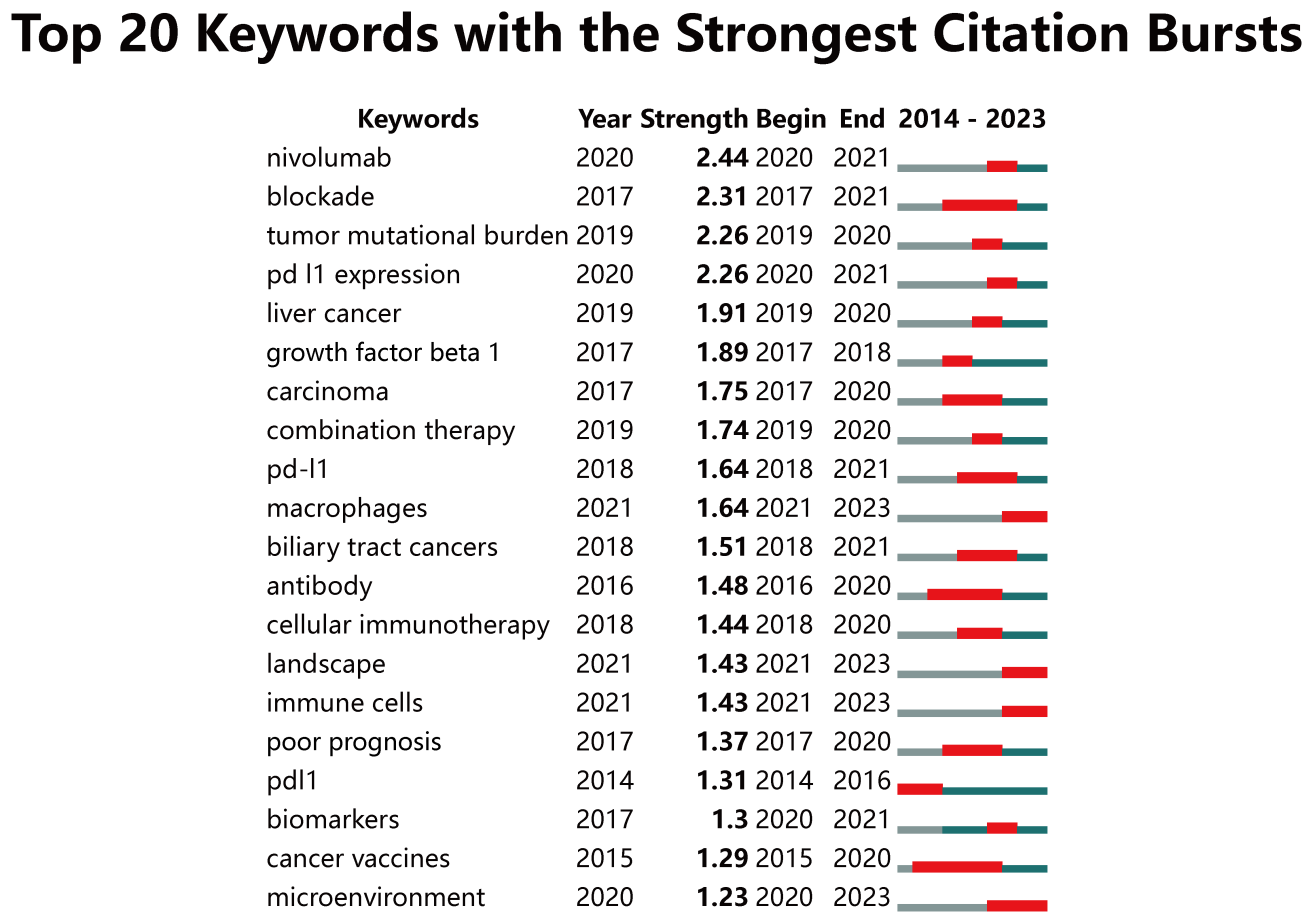
Keywords with the strongest citation bursts related to immunotherapy for CCA patients from 2014 to 2023. The blue line represents the time axis, with the red part indicating the start year, end year, and duration of the burst.

After eliminating meaningless keywords through the VOS viewer, we found that the most frequently occurring keywords included “chemotherapy,” “expression,” “gemcitabine,” “tumor microenvironment,” and “open-label” (**Supplementary Table S3**). The development of keywords over time can reflect the evolution of cutting-edge knowledge (24). We designed a timeline view using CiteSpace to display the keywords. The data can be roughly divided into 13 clusters. Among them, related hotspots such as tumor mutational burden, microenvironment, PD-L1 expression, nivolumab, combination therapy, targeted therapy, immune cells, and macrophages have been the primary areas of interest in CCA immunotherapy since 2019 (**Supplementary Figure S4**). A burst keyword is a keyword that has been widely cited over a period, and research frontiers can be identified by detecting burst keywords (25). We analyzed 20 keywords with the highest burst intensity from January 2014 to December 2023 (**Figure 8**). Among them, “nivolumab,” “blockade,” “tumor mutational burden,” and “PD-L1 expression” ranked at the top with the burst strength of 2.44, 2.31, 2.26 and 2.26, respectively. In addition, “microenvironment” (2020-2023), “immune cells” (2021-2023), and “macrophages” (2021-2023) burst continually till 2023 and have been emerging as current hotspots.

From 2014 to 2018, research on immunotherapy for CCA primarily focused on molecular mechanisms. Subsequently, ICIs and predictive biomarkers emerged as new focal points and experienced explosive growth between 2019 and 2021. Recently, the tumor microenvironment (TME) has become a prominent research hotspot from 2022 to 2023.

### 3.7 Analysis of ongoing immunotherapy clinical trials

Currently, numerous clinical trials are being carried out to explore the potential of immunotherapy for CCA (**Table 4**). The treatment combinations can be divided into three categories: ACT, cancer vaccine-based combination therapy and ICI combination therapy. Among them, ICI combination therapy is the most common treatment regimen.

**Table 4:** Ongoing clinical trials of immunotherapy for CCA.

NCT Number	Categories	Interventions	Primary Outcome
NCT03633773	Adoptive cell therapy	MUC-1 CART cell immunotherapy	DCR
NCT03820310		Autologous Tcm cellular immunotherapy	PFS
NCT03942328	Cancer vaccine combination therapy	DCVAC + EBRT	ORR+PFS
NCT06406816		Neoantigen Vaccine + Capecitabine	SP+AEs
NCT06490198		OBI-833/OBI-821 + GC	PFS
NCT04003636	ICI combination therapy	Pembrolizumab + GC	PFS+ORR
NCT04238637		Durvalumab + Tremelimumab	ORR
NCT04298008		Durvalumab + AZD6738	DCR
NCT04308174		Durvalumab + GC	OS
NCT04660929		Pembrolizumab + CT-0508	PFS+ORR
NCT05215665		Lenvatinib + GEMOX + Toripalimab	ORR
NCT05239169		Durvalumab + Tremelimumab + Capecitabine	RFS
NCT05655949		Durvalumab + GC + Yttrium-90	PFS
NCT05849480		Pembrolizumab + XELOX + CDX-1140	PFS+ORR
NCT06341764		Durvalumab + Tremelimumab + GC	DRR
NCT06375915		Durvalumab + GC + Yttrium-90	ORR

MUC-1, The transmembrane glycoprotein mucin 1; CART, Chimeric antigen receptor T cell; Tcm, Central memory T cells; DCVAC, Autologous dendritic cell-based vaccine; EBRT, External beam radiotherapy; GC, Gemcitabine plus Cisplatin; GEMOX, Gemcitabine plus Oxaliplatin; XELOX, Oxaliplatin plus Capecitabine; ORR, Objective response rate; OS, Overall survival; PFS, Progression-free survival; DCR, Disease control rate; SP, Safety parameters; AEs, Adverse events; DRR, Disease recurrence rate; RFS, Recurrence-free survival.

## 4 Discussion

To identify the current status and hotspots of CCA immunotherapy research worldwide, this study screened publications in the last decade related to CCA immunotherapy in the *WOS Core Collection* and conducted the first bibliometric analysis of this topic. The bibliometric analysis identified publication growth trends, the most active countries and institutions, journals and authors, top citations, and research keywords. The research focused on the field of CCA immunotherapy was observed to shift from “chemotherapy” and “immune checkpoint” to “immunotherapy combined therapy,” and current research frontiers are concentrated in the “microenvironment,” “immune cells,” and “macrophages.” Our study provides a novel and comprehensive knowledge map for CCA immunotherapy research and reveals the current state of research and trends, which can guide clinicians and researchers and identify directions for future research.

In this study, we found that China, the United States, and Thailand have made outstanding contributions to CCA immunotherapy research. The country with the greatest number of publications is China, while the United States has made the greatest contribution to CCA immunotherapy in terms of total citation frequency. The authors with the highest number of publications are from Thailand and China. Notably, this finding roughly coincides with the global epidemiological profile of CCA. The incidence of CCA ranges from 0.6/100,000 to 1/100,000 in the United States, 0.97/100,000 to 755/100,000 in China, and 85/100,000 in northeastern Thailand (26–28). In addition, although multiple risk factors for CCA have been identified, including cholestatic liver disease, cirrhosis, and biliary stones, there are significant differences in the predominant risk factors between Western and Asian countries (29). Primary sclerosing cholangitis is most frequently observed in Western countries such as the United States, whereas liver fluke infection is most common in Asian countries such as China and Thailand (30, 31). Overall, the United States dominates the field of CCA immunotherapy due to the advantages of advanced technology and numerous research institutions, while China and Thailand have increased their research investment because of the large number of patients. However, there remains a lack of in-depth cooperation between China, the United States, and Thailand. In the future, there is a need to fully utilize the technical and resource advantages of both Western and Asian countries to promote the progress of research in this field jointly.

The focus of research on CCA immunotherapy has shifted from “chemotherapy” and “immune checkpoint” to “immunotherapy combined therapy” over the last decade. Initially, the early stages of CCA immunotherapy research were focused on chemotherapy and immune checkpoint inhibitors (ICIs). In 2010, Juan Valle et al. discovered that the combination of cisplatin with gemcitabine offered significant survival benefits compared to gemcitabine alone without adding substantial toxicity (32). This finding established the theoretical foundation for the new chemotherapy regimen of gemcitabine plus cisplatin (GC) as a guideline-recommended first-line treatment for advanced CCA. However, even with this regimen, the median overall survival (mOS) remained limited to less than 12 months (32). Fortunately, ICIs, which are aimed at restoring the functionality of tumor-specific T cells to combat malignant tumors, have been shown to be effective in many types of cancer (33). However, immunosuppressants inevitably encountered challenges such as low single-drug response rates and resistance to long-term use. Therefore, the current phase of immunotherapy for CCA has involved exploring combinations of ICIs with chemotherapy (34). Treatment with PD-1/L1 inhibitors and gemcitabine/cisplatin (GC) has shown a greater objective response rate (ORR) and mOS in most CCA clinical studies than single immunosuppressive therapy. For example, Nabumab monotherapy had an ORR of 3.3% and an mOS of 5.2 months, while the Nabumab + GC combination had an ORR and an mOS of 36.7% and 15.4 months, respectively (35). Additionally, combinations of ICIs with other therapies, including targeted therapy, radiotherapy, T cell therapy, and cancer vaccines, are also being explored and have shown promising efficacy (7). For instance, the ORR and mOS of CCA patients treated with pembrolizumab alone were 5.8% and 7.4 months, whereas those treated with pembrolizumab in combination with levatinib were 25% and 11 months, respectively (36, 37). In conclusion, with increasing research on immunotherapy for CCA, combination therapy has led to improved long-term survival for patients with CCA.

Recent studies on immunotherapy for CCA have concentrated on the following three themes: “microenvironment,” “immune cells,” and “macrophages.” First of all, TME in CCA is highly heterogeneous and contains various immune cells, stromal cells, and endothelial cells, as well as proliferative factors (38). Based on the cellular composition of TME, CCA can be classified into four subtypes: immune-desert, immune-active, myeloid, and stromal (17). Remarkably, the stratification characterized by immune subtypes is of great value for the development of personalized treatment strategies. Certain drugs, such as chemotherapy agents, have the capacity to transform immune “cold” CCA into immune “hot” one, enhancing the tumor’s immunogenicity and leading to greater potential benefits from immunotherapy (39). In addition, Immune cells, such as tumor-infiltrating lymphocytes (TILs) and tumor-associated macrophages (TAMs), play crucial roles in the antitumor immune response. On one hand, CD4+ TILs are mainly found in the peritumoral region, whereas CD8+ TILs are predominantly prevalent in intratumoral CCA tissues (40). The distribution of these immune cells may be related to the immune surveillance of CCA and tumor escape mechanisms. Based on TILs, adoptive T cell therapy harvests patients’ tumor-infiltrating lymphocytes, which are then expanded and re-infused into patients (41). Nowadays, in several clinical trials, adoptive T cell therapy is used as a monotherapy or in combination with immune checkpoint inhibitors, aiming to evaluate their therapeutic potential in CCA (41). On the other hand, TAMs are classified into two categories: classically activated (M1) phenotype that is pro-inflammatory and antitumor, and alternatively activated (M2) phenotype that promotes tumor growth and immune suppression (42). TAMs secrete cytokines such as TNF-α, TGF-β, and IL6, which regulate the CCA microenvironment and promote epithelial-mesenchymal transition, tumor growth, and metastasis (43). To enhance potential therapeutic efficacy in CCA, a series of clinical trials are conducted through emerging strategies for targeting TAMs in CCA, such as inhibiting the transition of monocytes to M2 TAMs, remodeling M2 TAMs to M1 TAMs, eliminating specific pre-tumoral tissue-resident macrophages, and blocking the communication between M2 TAMs and cancer cells (44). Additionally, TME is also closely related to the response to immunotherapy in CCA patients. Several studies have shown that CCA patients with abundant CD8+ T cell infiltration, significant PD-L1 expression, high-level microsatellite instability (MSI-H) or mismatch repair deficiency (dMMR), and high tumor mutational burden (TMB) exhibit better sensitivity to immunotherapy (45, 46). Hence, CD8+ T cell infiltration, PD-L1 expression, MSI, and TMB are potential biomarkers for predicting response to immunotherapy (12). Taken together, high heterogeneity and complexity related to the TME appear to be linked to treatment failures involving immunotherapy for CCA (20, 47, 48). Therefore, future studies on the TME and its immune cells will provide potential new avenues for CCA immunotherapy.

Currently, ICI combination therapy, including durvalumab and pembrolizumab, is the leading immunotherapeutic approach for CCA. The findings from the TOPAZ-1 study revealed that the group treated with a combination of durvalumab, gemcitabine, and cisplatin chemotherapy achieved an impressive ORR of 73.4%. Moreover, this group exhibited a significantly longer mOS of 18.1 months compared to 11.7 months in the chemotherapy-only group (47). The KEYNOTE-966 study showed that the mOS was 12.7 months in the pembrolizumab in combination with gemcitabine and cisplatin group versus 10.9 months in the group receiving gemcitabine and cisplatin alone for patients with advanced CCA (49). These results provide compelling support for the adoption of ICI combination therapy in the treatment of advanced CCA. Therefore, in 2022, the combination of durvalumab in combination with GC received FDA approval for patients with locally advanced or metastatic CCA, marking a breakthrough for the combination of immunotherapy with chemotherapy as a first-line treatment for CCA (50). Additionally, researchers are exploring other immune checkpoints, such as lymphocyte activation gene 3, T-cell immunoglobulin and mucin domain-3, and T-cell immunoreceptor with immunoglobulin and ITIM domains protein (TIGIT) (51). Notably, the anti-TIGIT antibody has gained increasing attention and is expected to be the next-generation ICI (52). In summary, it is anticipated that ICI-based combination therapy will continue to play a crucial role as an immunotherapy strategy for CCA in the foreseeable future.

In the future, the goal of immunotherapy for CCA is to advance precision medicine and personalized therapeutics. First of all, although immunotherapy has been included in several guidelines, such as “British Society of Gastroenterology guidelines for the diagnosis and management of cholangiocarcinoma,” ESMO clinical practice guideline for diagnosis, treatment and follow-up of biliary tract cancer,” and “EASL-ILCA clinical practice guidelines on the management of intrahepatic cholangiocarcinoma,” its indication seems to be slightly conservative and varies in different populations (53). Therefore, more large-scale, international multicenter, randomized controlled clinical trials are essential for providing further robust evidence to highlight its pivotal role in CCA treatment. In addition, considering the level of immune cell infiltration in the tumor tissue, TME of CCA can be classified into “inflamed” or “non-inflamed,” which is closely linked to specific molecular subtypes of CCA (54). Unfortunately, most CCA cases displayed a non-inflamed TME characterized by a significant absence of effector T cells, rendering ICIs ineffective in such cases. However, patients with intrahepatic cholangiocarcinoma harbor frequent genetic alterations such as isocitrate dehydrogenase (IDH)-1 (20.6–29.1%) and -2 (2.5–4.4%) mutations and fibroblast growth factor receptor 2 (FGFR2) fusions (10–15%), which can be treated with IDH1/2 or FGFR2 inhibitors (55, 56). Hence, there is still a compelling need to explore the molecular typing of CCA using DNA- or RNA-based next-generation sequencing to facilitate synergistic immunotherapy of CCA. Moreover, for patients without favorable benefits from ICIs, further studies are warranted to provide alternative therapeutic modalities. Various forms of T cell therapies, like chimeric antigen receptor (CAR) T cell therapy, T cell receptor (TCR) therapy, adoptive T cell therapy, and TIL therapy, have opened a new avenue for improving outcomes in CCA (57, 58). Oncolytic adenovirus system-based photodynamic immunotherapy may offer a promising immunotherapeutic strategy for CCA (59). Finally, it is crucial to develop novel biomarkers to identify the patient population that would benefit from immunotherapy more accurately. Recently, a growing number of studies have been performed to better predict the clinical response of CCA to immunotherapy using cutting-edge techniques, including artificial Intelligence-based macrophages or TILs signatures as well as gut microbiota and metabolites signatures (60–63).

This study has certain limitations. Firstly, due to the limitations of visual analysis software, it is difficult to merge and analyze data from different databases (e.g., PubMed or Scopus). Therefore, we only searched the WoSCC database, which may omit some relevant studies. However, it is worth noting that WoSCC is the most commonly used database for scientometrics research. In addition, limiting the analysis to English-language studies may restrict insights, particularly given CCA’s high prevalence in Asia, where significant studies may be published in non-English journals. This exclusion could indeed impact the geographic and cultural representation of our findings, potentially overlooking or underestimating related region-specific factors and perspectives. In the future, we can broaden the scope of our data by including more databases and publications in other languages.

## 5 Conclusion

This comprehensive bibliometric analysis provides a complex overview of the current state of research regarding CCA immunotherapy over the past decade. The current research focus has shifted from “chemotherapy” and “immune checkpoint” to “immunotherapy combined therapy.” ICIs combined with chemotherapy have become the dominant combination strategy. The combination of ICIs with other therapeutic modalities, including targeted therapy, radiotherapy, adoptive T cell therapy, and cancer vaccines, is still being explored. In addition, “microenvironment,” “immune cells,” and “macrophages” have become the frontiers of CCA immunotherapy. In-depth research on TME will help us promote personalized precision treatment of CCA. Therapies targeting TILs or TAMs in TME are expected to bring a new round of breakthroughs in this field. Overall, this study provides a comprehensive knowledge map of CCA immunotherapy research, and identifies hotspots and trends, which can be utilized as a reference and provide direction for future research.

## Data Availability

The raw data supporting the conclusions of this article will be made available by the authors, without undue reservation.
