# Supplementary material for: Research hotspots and trends in immunotherapy for cholangiocarcinoma: a bibliometric analysis (2014-2023)
Source: Front Immunol. 2024 Nov 26;15:1436315. doi: 10.3389/fimmu.2024.1436315 (PMC11628549; doi:10.3389/fimmu.2024.1436315)
Supplement: Supplementary file 1 [file DataSheet1.docx]

***Supplementary Material***

**Supplementary Figure S1** A visualization of co-cited authors related to immunotherapy for CCA.

**Supplementary Figure S2** A visualization of author citation analysis.

**Supplementary Figure S3** A visualization of co-cited references related to immunotherapy for CCA.

**Supplementary Figure S4** Timeline view of keywords related to immunotherapy for CCA.

**Supplementary Table S1** The top 10 prolific authors.

**Supplementary Table S2** The top 10 co-cited references.

**Supplementary Table S3** The top 10 most frequent keywords.


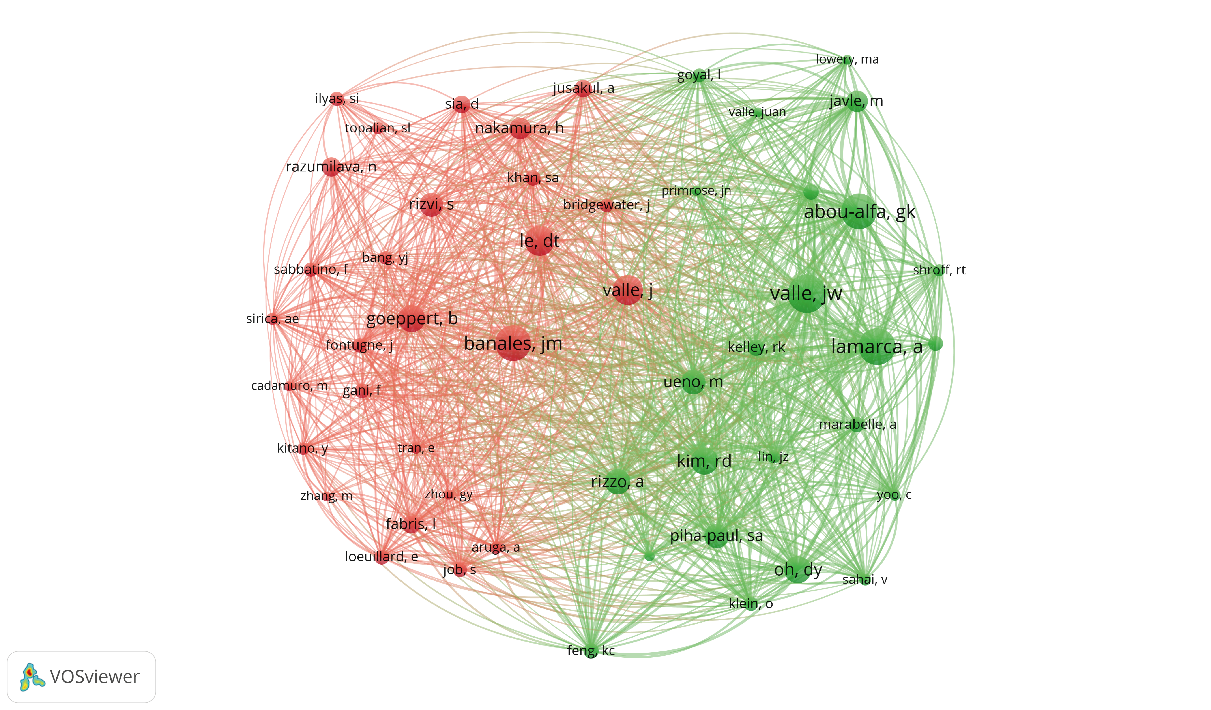


**Supplementary Figure S1** A visualization of co-cited authors related to immunotherapy for CCA.


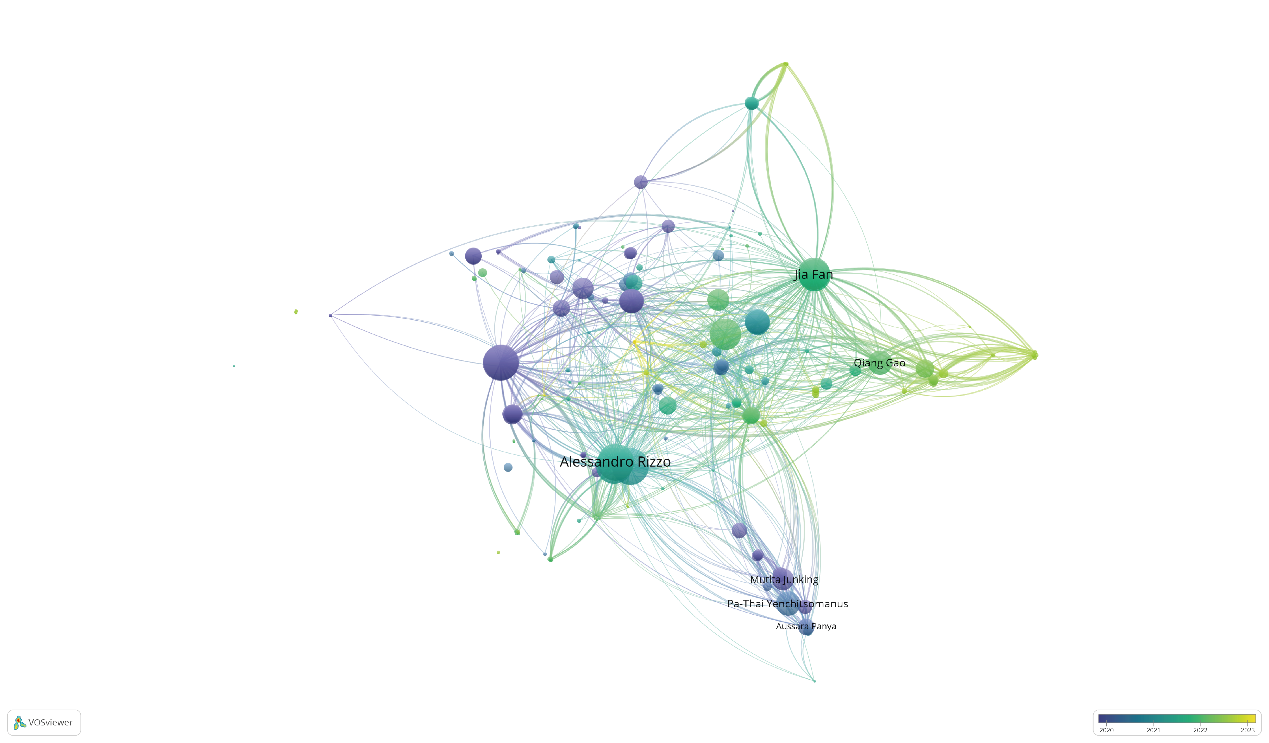


**Supplementary Figure S2** A visualization of author citation analysis.


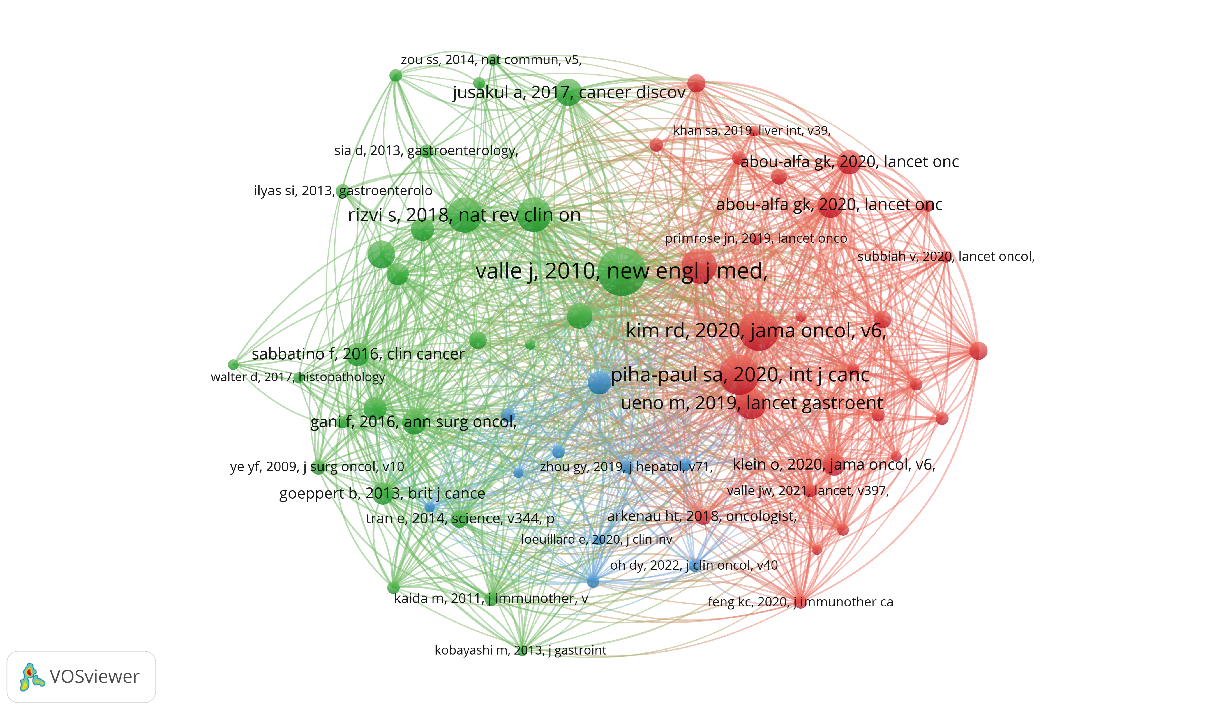


**Supplementary Figure S3** A visualization of co-cited references related to immunotherapy for CCA.


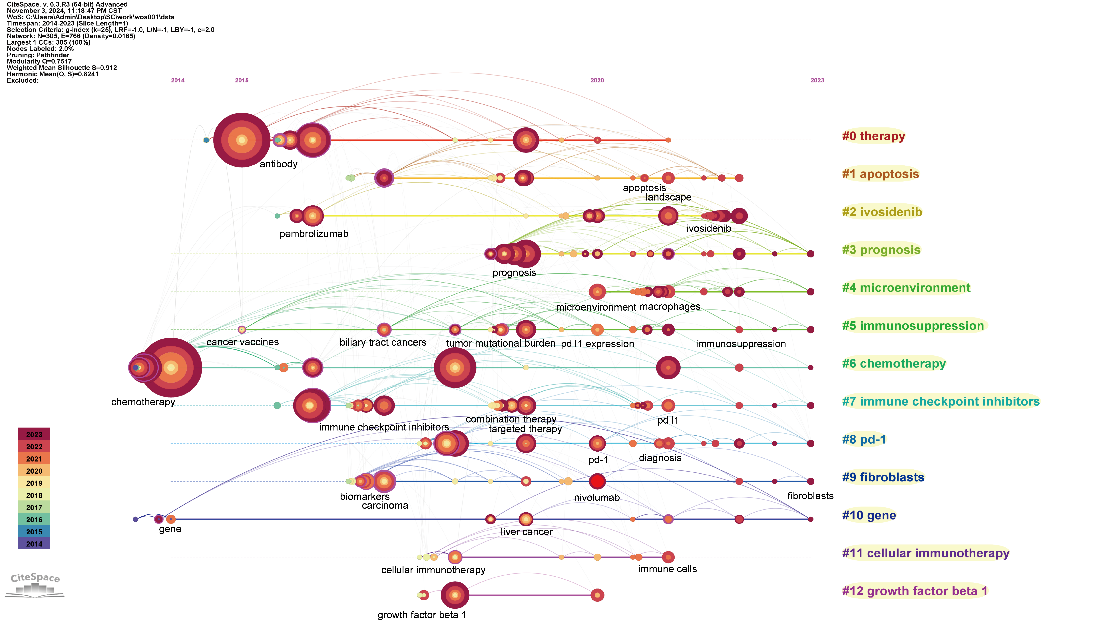


**Supplementary Figure S4** Timeline view of keywords related to immunotherapy for CCA.

**Supplementary Table S1** The top 10 prolific authors.

| Rank | Author | Country | Count | Citations | Citation per articles |
| --- | --- | --- | --- | --- | --- |
| 1 | Jia Fan | China | 9 | 283 | 31.44 |
| 2 | Jian Zhou | China | 9 | 283 | 31.44 |
| 3 | Pa-Thai Yenchitsomanus | Thailand | 9 | 215 | 23.89 |
| 4 | Alessandro Rizzo | Italy | 8 | 330 | 41.25 |
| 5 | Giovanni Brandi | Italy | 7 | 327 | 46.71 |
| 6 | Angela Dalia Ricci | Italy | 7 | 322 | 46.00 |
| 7 | Qiang Gao | China | 7 | 213 | 30.43 |
| 8 | Aussara Panya | Thailand | 7 | 144 | 20.57 |
| 9 | Mutita Junking | Thailand | 6 | 192 | 32.00 |
| 10 | Nunghathai Sawasdee | Thailand | 6 | 82 | 13.67 |

**Supplementary Table S2** The top 10 co-cited references.

| Title | First  Author | Journals | IF（2023） | Year | Citations |
| --- | --- | --- | --- | --- | --- |
| Cisplatin plus gemcitabine versus gemcitabine for biliary tract cancer | Juan Valle | The New England journal of medicine | 96.2 | 2010 | 78 |
| A Phase 2 Multi-institutional Study of Nivolumab for Patients With Advanced Refractory Biliary Tract Cancer | Richard D Kim | JAMA Oncology | 22.5 | 2020 | 64 |
| Efficacy and safety of pembrolizumab for the treatment of advanced biliary cancer: Results from the KEYNOTE-158 and KEYNOTE-028 studies | Sarina A Piha-Paul | International journal of cancer | 5.7 | 2020 | 64 |
| Cholangiocarcinoma 2020: the next horizon in mechanisms and management | Jesus M Banales | Nature Reviews Gastroenterology and Hepatology | 45.9 | 2020 | 56 |
| Cholangiocarcinoma - evolving concepts and therapeutic strategies | Sumera Rizvi | Nature reviews. Clinical oncology | 81.1 | 2018 | 56 |
| Genomic spectra of biliary tract cancer | Hiromi Nakamura | Nature genetics | 31.7 | 2015 | 55 |
| Nivolumab alone or in combination with cisplatin plus gemcitabine in Japanese patients with unresectable or recurrent biliary tract cancer: a non-randomised, multicentre, open-label, phase 1 study | Makoto Ueno | The Lancet Gastroenterology & Hepatology | 30.9 | 2019 | 51 |
| Cholangiocarcinoma | Nataliya Razumilava | Lancet | 98.4 | 2014 | 45 |
| Whole-Genome and Epigenomic Landscapes of Etiologically Distinct Subtypes of Cholangiocarcinoma | Apinya Jusakul | Cancer discovery | 29.7 | 2017 | 44 |
| Pemigatinib for previously treated, locally advanced or metastatic cholangiocarcinoma: a multicentre, open-label, phase 2 study | Ghassan K Abou-Alfa | Lancet Oncol | 41.6 | 2020 | 42 |

**Supplementary Table S3** The top 10 most frequent keywords.

| Rank | Keyword | Count | Centrality |
| --- | --- | --- | --- |
| 1 | chemotherapy | 49 | 0.06 |
| 2 | expression | 48 | 0.04 |
| 3 | gemcitabine | 38 | 0.18 |
| 4 | tumor microenvironment | 30 | 0.00 |
| 5 | prognosis | 27 | 0.01 |
| 6 | therapy | 25 | 0.00 |
| 7 | multicenter | 23 | 0.01 |
| 8 | pd-l1 | 23 | 0.10 |
| 9 | pembrolizumab | 21 | 0.05 |
| 10 | immune checkpoint inhibitors | 19 | 0.00 |
